# Effect of a Quality Improvement Initiative on Procedural Abortion Pain and Anxiety Using a Standardized Patient-Focused Sedation Options Counseling Guide

**DOI:** 10.7759/cureus.97850

**Published:** 2025-11-26

**Authors:** Stephanie L Small, Jonathan F Emery, Vicki Hart, Elzerie de Jager

**Affiliations:** 1 Healthcare Delivery, Planned Parenthood of Northern New England, Topsham, USA; 2 Biomedical Statistics, University of Vermont, Burlington, USA; 3 Public Health Science and Research Division, DLH Corporation, Silver Spring, USA; 4 Medicine, University of Vermont, Burlington, USA

**Keywords:** abortion, anxiety, counseling, pain, sedation

## Abstract

Objective: To evaluate the effect of a quality improvement initiative on procedural abortion pain and anxiety using a standardized patient-focused sedation options counseling guide.

Study design: We developed and implemented a patient-focused sedation options counseling guide for use across three reproductive health centers in Maine, New Hampshire, and Vermont. To evaluate the effect of the initiative, pain (0-10) and anxiety (0-5) scores were abstracted from the electronic health record for all patients who obtained procedural abortions with moderate sedation through 18 weeks of gestation (N = 362). A six-month pre/post implementation analysis was conducted examining pain and anxiety scores. To analyze differences in pain and anxiety while controlling for covariates, a hurdle model was fit with maximum intraoperative pain scores, and Poisson regression was fit with maximum full procedure anxiety scores as the outcomes of interest.

Results: The proportion of patients reporting no pain increased from 13.0% to 22.7% with the use of the counseling guide (p = 0.03). The median maximum intraoperative pain score decreased from 5 to 4. In the unadjusted model, use of the counseling guide decreased the odds of patients reporting any intraoperative pain by 49% (odds ratio (OR): 0.51, 95% confidence interval (CI): 0.27-0.91). The odds ratio was similar after adjustment for covariates (OR: 0.52, 95% CI: 0.26-1.04). Use of the counseling guide showed no significant effect on anxiety.

Conclusion: We saw a reduction in procedural abortion pain after the implementation of the counseling guide. This adjunct to established pain management options is a simple improvement on current practice.

## Introduction

Problem

Health centers use various sedation options to manage pain and anxiety for patients undergoing procedural abortions. While many studies have evaluated the direct effect of interventions on pain and anxiety during abortion procedures [1-3], to our knowledge, no studies have evaluated the effect of sedation options counseling on procedural pain and anxiety. Without a patient-focused framework to standardize the counseling, the counseling could be subject to variability among individual staff members and influenced by unconscious bias held by health care providers [4]. Developing a quality improvement initiative to assist staff in providing sedation options counseling while working within the context of a history of pain disparities in healthcare [5] and in support of health equity [6], we wondered if standardizing counseling to be more patient-focused would provide patients with decision-making control that may ease perceived procedural pain and anxiety, and then designed a study to evaluate it.

Available knowledge

Many individual variables can affect a patient’s experience of abortion pain but are not directly modifiable by health centers. These variables include patient depression, anxiety, anticipation of pain, nulliparity [3], and gestational age greater than 10 weeks [7]. While health care providers cannot influence these individual variables, they can use procedural or pharmacologic interventions, including paracervical blocks [1], non-steroidal anti-inflammatory drugs [3], and intravenous fentanyl [2], as effective options for reducing pain in patients receiving abortions. Health centers often use protocols to provide guidelines for the administration of procedural and pharmacologic interventions. However, attempts to further standardize medication administration and reduce practice variation by using a patient-centered algorithm for dosing did not result in improved procedural abortion pain [8]. Attempts to use nonpharmacological or nonprocedural interventions to reduce pain, including support interventions, music therapy, acupoint stimulation, and hypnotic analgesia, also have not shown consistent benefit [9].

Prior to procedures, health centers often provide counseling for patients about what pain control and sedation options are available. Practice guidelines (not specific to abortion) recommend “Before the procedure, inform patients or legal guardians of the benefits, risks, and limitations of moderate sedation or analgesia and possible alternatives and elicit their preferences” [10] based on expert opinion. However, guidelines do not provide evidence-based recommendations on how best to elicit patient preferences to provide informed consent. Furthermore, while informed consent is standard practice for sedation administration, there are no investigations of whether the process for eliciting patient preference for the type of sedation influences the patient’s experience of pain or anxiety.

Rationale

In a randomized controlled trial involving 756 family planning patients, researchers found that implementing patient-focused counseling practices during contraception visits significantly enhanced the patient experience during the visits [11]. The patient-focused contraceptive counseling used 10 practices to support patient decision-making, such as demonstrating trustworthiness, expertise, and accessibility as a counselor, addressing side effects, simplifying choices, and addressing how a method fits with the patient’s lifestyle. Patients who received the patient-focused contraceptive counseling had higher satisfaction with the counseling. We adapted this evidence-based counseling guide to develop a framework for counseling on sedation options, aiming to provide a more standardized and patient-focused experience.

Specific aims

The project was intended as a quality improvement effort to provide patient-focused sedation options counseling in our health centers by developing a counseling guide for staff use. We examined the impact of the initiative using available data, specifically pain and anxiety scores in patients receiving moderate sedation. The first specific aim was to assess the influence of our patient-focused counseling initiative on patient experience of pain during abortion procedures. The second specific aim was to assess the influence of our patient-focused counseling intervention on patient experience of anxiety during abortion procedures.

This article was previously presented as a poster presentation at the National Abortion Federation’s 47th Annual Meeting on April 29-30, 2024.

## Materials and methods

Context

Planned Parenthood of Northern New England (PPNNE) provides over 52,000 visits annually, and 7% of visits are abortion related [12]. In Portland, Maine, Manchester, New Hampshire, and Burlington, Vermont, nurse practitioners, certified nurse midwives, physician assistants, and physicians perform procedural abortions and administer sedation. Sedation includes the administration of analgesia and sedating agents. The options provided include the following: (1) no sedation (defined as no medication other than ibuprofen, tramadol, and a paracervical anesthetic block to reduce pain); (2) minimal sedation, which includes oral medications (specifically acetaminophen with codeine or pregabalin to reduce pain and lorazepam to reduce anxiety); and (3) moderate sedation, which includes intravenous medications (specifically fentanyl to reduce pain and midazolam to reduce anxiety). There is no difference in patient cost among the sedation options, so patients may choose their option without consideration of financial impact. Prior to sedation administration and abortion procedures, health care assistants talk with patients individually to elicit patient preference for sedation options and provide informed consent. While the informed consent document for signature is standardized across the organization, the counseling process to elicit patient preference for sedation options had no framework or guidance on how best to discuss sedation options with patients. We implemented a quality improvement initiative to help support staff providing sedation options counseling, standardizing the focus on patient priorities.

Intervention

Having successfully trained staff in the past to use an evidence-based tool [11] to guide and standardize contraceptive options counseling at PPNNE, the training team recognized the benefit of standardizing the approach to counseling around decision-making and elected to apply a similar approach to sedation options counseling. Using the evidence-based contraceptive options counseling tool [11] as a basis, a PPNNE interdisciplinary team developed a new, unpublished sedation options counseling guide (Appendices) with the goal of centering patient priorities and reducing the possibility of staff bias. The training team then conducted a 20-minute training session during the virtual June 2021 quarterly meeting for all staff at all health centers providing abortions. A PowerPoint presentation (Microsoft Corporation, Redmond, Washington) provided an overview of the guide development process, reviewed the importance of centering patients and their concerns, and outlined the updated workflow to include the new guide. The Standardized Patient-Focused Sedation Options Counseling Guide, which lists the sedation options and common questions about sedation, was presented. Staff were given examples of how to use the guide to ask about which of the sedation considerations were priorities for the individual patient and then focus the discussion on those priorities. Staff had an opportunity to ask questions at the presentation. On July 14, 2021, all three health centers began using the sedation options counseling guide. At the next quarterly meeting in September 2021, PPNNE checked back with staff about the utility of the guide after three months of use with an open forum for feedback and discussion.

Study of the intervention

Interested in evaluating the new guide as part of continuous quality improvement in health care provision, we chose a pragmatic design to make use of existing data. Because staff were already collecting and documenting pain scores (0-10) and anxiety scores (0-5) preoperatively, intraoperatively, and postoperatively as part of procedural workflows for patients receiving moderate sedation, we compared pain and anxiety scores for six months before the quality improvement initiative (January 13, 2021, to July 13, 2021) to six months after we began using the patient-focused counseling guide (July 14, 2021, to January 14, 2022) to evaluate any effect the quality improvement initiative had on pain and anxiety scores. Staff were advised not to make any changes to the guide during the six-month post-initiative study period to ensure consistency in measurement. All staff were trained on the new counseling guide, but the data collection of pain and anxiety scores remained unchanged. We limited analysis to patients receiving moderate sedation to make use of existing pain and anxiety scores (pain and anxiety scores were not available for the oral sedation or no sedation options) and to eliminate any effect that different sedation options may have on procedural pain and anxiety.

Measures

We used pain and anxiety measures from our existing workflow and electronic health record (EHR). Clinicians and health care assistants verbally asked patients to report their level of pain and anxiety preoperatively, intraoperatively, and postoperatively and documented the scores in the EHR in real time. Pain scores, assessed using the numeric rating scale (NRS) as a scale from 0 (no pain) to 10 (worst possible pain), were extracted from the EHR for analysis. The NRS is a validated tool commonly used to assess pain [13]. To assess anxiety, we also used a numeric scale: 0 (no anxiety) to 5 (very high anxiety). Although this scale is not a validated anxiety measure, it is similar to the validated [14] Visual Analogue Scale for Anxiety (VAS-A). These data are routinely obtained during abortion procedures and captured in the EHR, providing an accessible measure to compare pre-initiative and post-initiative results.

Analysis

**Figure 1:**
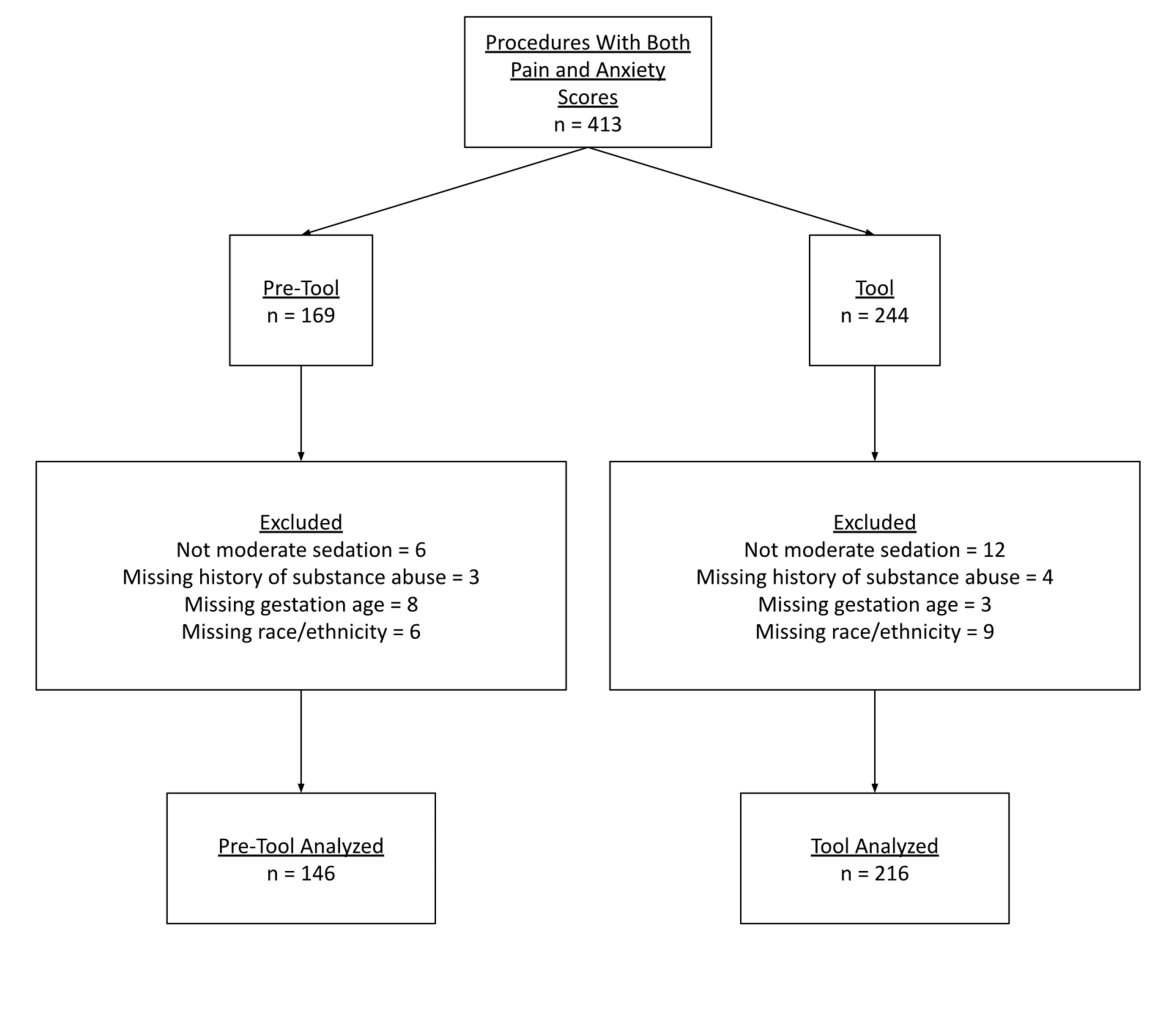
Cohort Diagram

Out of 413 procedures with both pain and anxiety scores (169 pre-initiative and 244 post-initiative), we excluded any procedures in which moderate sedation was not administered (6 pre-initiative and 12 post-initiative) and any procedures with missing covariate data (17 pre-initiative and 16 post-initiative) (Figure 1). This left 362 procedures, with 146 in the pre-initiative group and 216 in the post-initiative group.

**Figure 2:**
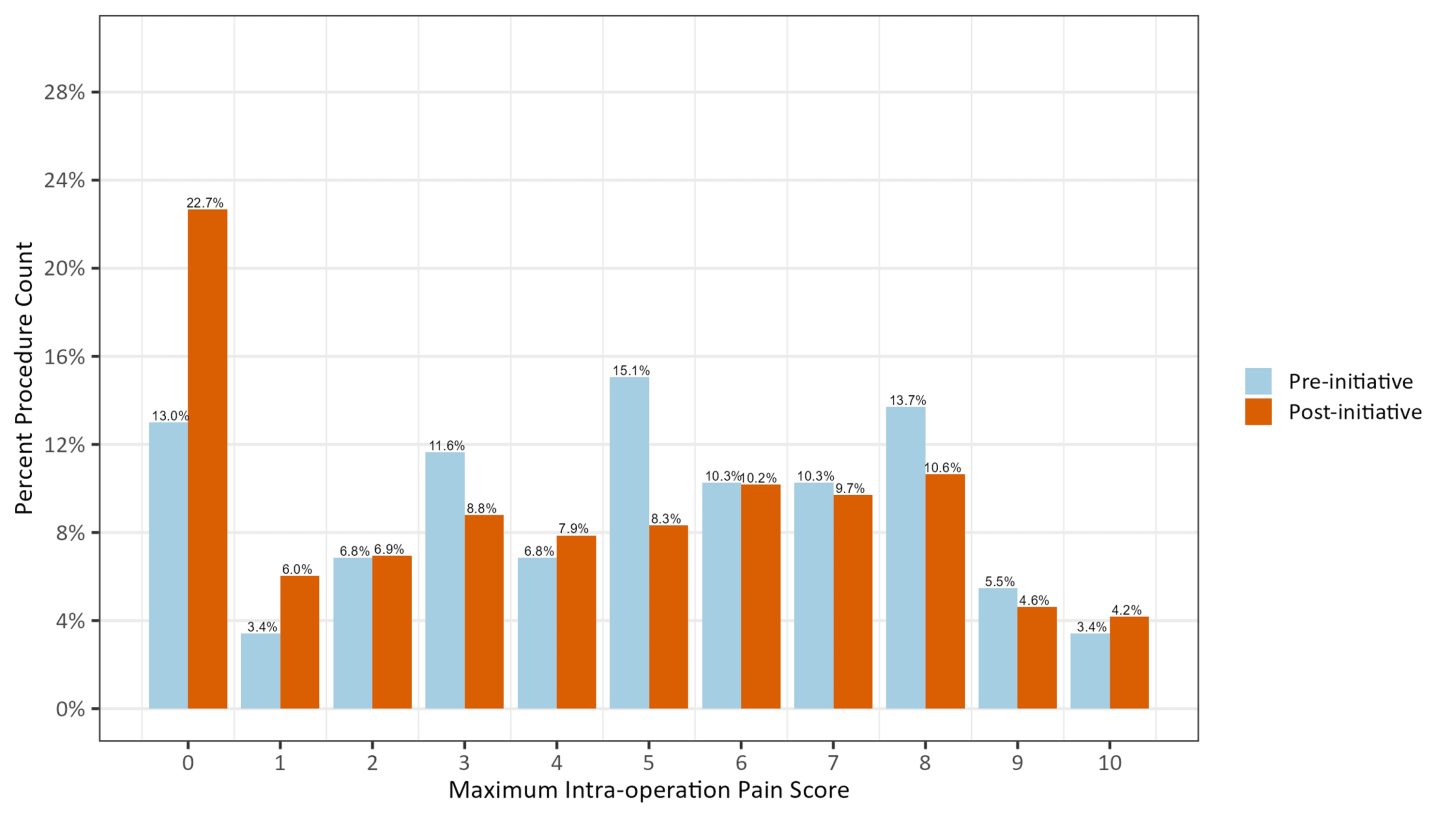
Distribution of Percent Procedure Count Along Each Maximum Intraoperative Pain Score Separated by Pre-initiative and Post-initiative The data are presented as a percentage of the total procedure count for each possible maximum intraoperative pain score from 0 to 10.

For pain, the maximum intraoperative scores were selected as the outcome of interest. This is due to their straightforward clinical interpretation, which is measuring the level of pain at the most uncomfortable moment during the procedure. Given that the maximum intraoperative pain scores exhibited a highly right-skewed distribution with a substantial number of zero scores (Figure 2), a hurdle model was chosen. This model divides the analysis into two main components. Firstly, the scores were transformed into a binary outcome, where zero scores were coded as zero (no pain) and non-zero scores were coded as one (any pain). Logistic regression was performed to determine whether there was a significant difference in the proportion of patients reporting no pain versus any pain between the pre-initiative and post-initiative groups. Secondly, a Poisson regression was fit among all non-zero scores to evaluate whether there was a significant difference in scores among those who provided at least one non-zero intraoperative pain score between the pre-initiative and post-initiative groups.

**Figure 3:**
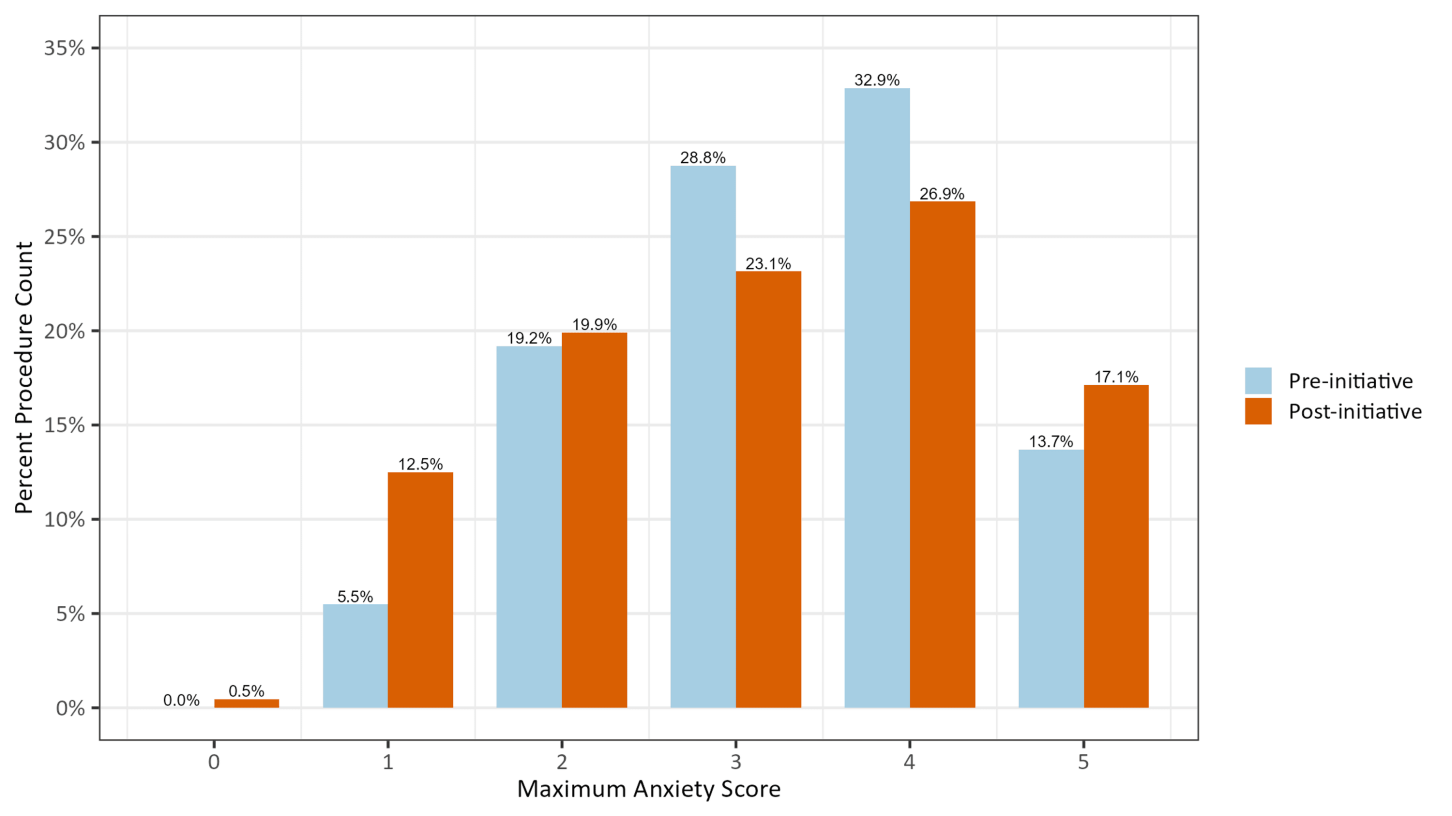
Distribution of Percent Procedure Count Along Each Maximum Full Procedure Anxiety Score Separated by Pre-initiative and Post-initiative The data are presented as a percentage of the total procedure count for each possible maximum intraoperative anxiety score from 0 to 5.

For anxiety assessment, the maximum full procedure scores were chosen to be the outcome of interest. This is due to their straightforward clinical interpretation, which measures the level of anxiety at the most anxious moment throughout the visit. The anxiety scores did not exhibit a strong skew (Figure 3), so a Poisson regression was fit among all scores, including zero.

Covariates were selected based on their potential relationship with intraoperative pain or full procedure anxiety and their availability within the EHR. Covariates included demographics (age, race or ethnicity defined by and collected for Title X reporting, and insurance type), procedure specifics (dosage of fentanyl, dosage of midazolam, and the total number of intraoperative pain or full procedure anxiety scores), and medical history variables (substance use disorder, anxiety, gestational age, and gravida). The total number of intraoperative pain scores and the total number of full procedure anxiety scores were included to account for variation in accuracy between groups. For example, a patient who has reported five intraoperative pain scores will have a maximum score closer to representing the true most painful moment when compared to a patient with only two intraoperative pain scores.

Statistical analyses were conducted using R version 4.2.3 (R Foundation for Statistical Computing, Vienna, Austria), along with the pscl package version 1.5.5.1 (Political Science Computational Laboratory, Stanford University, California).

## Results

Participant demographics in the pre-initiative and post-initiative groups are shown in Table 1. There was a significant difference in the history of anxiety (p = 0.042). No other significant differences in covariates were detected between groups.

**Table 1:** Characteristics of the Study Population Overall and by Pre-initiative and Post-initiative *P-values are calculated using the t-test for numeric variables **P-values are calculated using the chi-squared test for binary and categorical variables. Statistical significance was set at p<0.05.

Characteristics	Category	Overall (N = 362)	Pre-initiative (N = 146)	Post-initiative (N = 216)	Test Statistic	P-value
Age (in years), mean (SD)		26.5 (6.20)	26.9 (6.45)	26.2 (6.02)	1.12*	0.265
Gestational age (in days), mean (SD)		75.1(26.6)	75.8 (26.1)	74.6 (26.9)	0.449*	0.654
Total fentanyl (in µg), mean (SD)		126 (50.0)	121 (49.0)	129 (50.4)	-1.59*	0.112
Total midazolam (in mg), mean (SD)		2.39 (0.979)	2.49 (1.01)	2.33 (0.959)	1.49*	0.137
Number of pain scores, mean (SD)		3.75 (1.63)	3.78 (1.55)	3.73 (1.69)	0.286*	0.775
Number of anxiety scores, mean (SD)		5.72 (2.35)	5.52 (2.21)	5.85 (2.45)	-1.32*	0.187
Race and ethnicity, n (%)	Asian or Pacific Islander	10 (2.8%)	3 (2.1%)	7 (3.2%)	0.985**	0.964
	Hispanic	38 (10.5%)	16 (11.0%)	22 (10.2%)		
	Multiracial	14 (3.9%)	5 (3.4%)	9 (4.2%)		
	Non-Hispanic Black	21 (5.8%)	9 (6.2%)	12 (5.6%)		
	Non-Hispanic White	271 (74.9%)	109 (74.7%)	162 (75.0%)		
	Other	8 (2.2%)	4 (2.7%)	4 (1.9%)		
Site, n (%)	Burlington, VT	89 (24.6%)	32 (21.9%)	57 (26.4%)	1.13**	0.567
	Manchester, NH	149 (41.2%)	64 (43.8%)	85 (39.4%)		
	Portland, ME	124 (34.3%)	50 (34.2%)	74 (34.3%)		
Insurance, n (%)	Private	82 (22.7%)	31 (21.2%)	51 (23.6%)	5.05**	0.080
	Public	78 (21.5%)	24 (16.4%)	54 (25.0%)		
	Self-Pay	202 (55.8%)	91 (62.3%)	111 (51.4%)		
History of substance abuse disorder, n (%)	No	311 (85.9%)	121 (82.9%)	190 (88.0%)	1.47**	0.226
	Yes	51 (14.1%)	25 (17.1%)	26 (12.0%)		
History of anxiety, n (%)	No	324 (89.5%)	137 (93.8%)	187 (86.6%)	4.15**	0.042
	Yes	38 (10.5%)	9 (6.2%)	29 (13.4%)		
Gravida, n (%)	1	128 (35.4%)	51 (34.9%)	77 (35.6%)	1.44**	0.487
	2	64 (17.7%)	22 (15.1%)	42 (19.4%)		
	>2	170 (47.0%)	73 (50.0%)	97 (44.9%)		

The proportion of patients reporting no pain increased from 13.0% to 22.7% after we implemented the initiative (p = 0.03) (Table 2). The median maximum intraoperative pain score decreased from 5 to 4.4.

**Table 2:** Maximum Pain and Anxiety Scores by Pre-initiative and Post-initiative The data are presented as N(%) for no pain versus any pain along with median (Q1-Q3) for both maximum pain and anxiety scores. The P-value was calculated using the chi-squared test.

Maximum Score		Pre-initiative (N=146)	Post-initiative (N=216)	Test Statistic: Chi-Squared	P-value
Pain	No pain; n (%)	19 (13.0%)	49 (22.7%)	4.73	0.030
	Any pain; n (%)	127 (87.0%)	167 (77.3%)		
	Median (Q1-Q3)	5 (3-7)	4 (1-7)		
Anxiety	Median (Q1-Q3)	3 (3-4)	3 (2-4)		

In the unadjusted logistic regression model, the initiative decreased the odds of patients reporting any intraoperative pain by 49% (OR: 0.51, 95% CI: 0.27-0.91). The odds ratio was similar after adjustment for covariates, although the 95% confidence interval was wider (OR: 0.52, 95% CI: 0.26-1.04). We controlled for covariates and checked variance inflation factors to confirm no significant multicollinearity (Table 3). After controlling for covariates and confirming no significant multicollinearity, Poisson regression among patients who reported at least one non-zero intraoperative pain score showed no significant difference in maximum intraoperative pain scores between the pre-initiative and post-initiative groups (Table 4).

**Table 3:** Comparisons of Pain Versus No Pain Among Patients Pre-initiative and Post-initiative (N = 362) Model 1: Unadjusted Logistic regression comparing any Pain with No Pain. Model 2: Logistic regression comparing any Pain to No Pain adjusted for age, race/ethnicity, substance use, insurance, history of anxiety, gestational age, gravida, number of scores, and total fentanyl administered.

Model	Initiative	N	Odds Ratio	95% Confidence Interval
Model 1: Unadjusted	Pre-initiative	146	Ref	-
	Post-initiative	216	0.51	0.27-0.91
Model 2: Adjusted	Pre-initiative	146	Ref	-
	Post-initiative	216	0.52	0.26-1.04

**Table 4:** Comparisons of Pain Scores Among Patients Pre-initiative and Post-initiative (N = 294) Model 1: Unadjusted Poisson regression for maximum intraoperative pain among patients who reported pain during procedures. Model 2: Poisson regression for maximum intraoperative pain among patients who reported pain during procedures, adjusted for age, race/ethnicity, substance use, insurance, history of anxiety, gestational age, gravida, number of scores, and total Fentanyl administered.

Model	Initiative	N	Risk Ratio	95% Confidence Interval
Model 1: Unadjusted	Pre-initiative	127	Ref	-
	Post-initiative	167	0.97	0.88-1.08
Model 2: Adjusted	Pre-initiative	127	Ref	-
	Post-initiative	167	0.95	0.86-1.05

The median maximum full procedure anxiety score remained 3 after the introduction of the initiative (Table 2). After controlling for covariates and confirming no significant multicollinearity, Poisson regression showed no significant difference in maximum full procedure anxiety scores between the pre-initiative and post-initiative groups (Table 5).

**Table 5:** Poisson Regression to Detect Differences in Maximum Full Procedure Anxiety Score During Abortion Procedures (N = 362) Model 1: Unadjusted Poisson regression for maximum intraoperative pain among patients who reported pain during procedures. Model 2: Poisson regression for maximum intraoperative pain among patients who reported pain during procedures, adjusted for age, race/ethnicity, substance use, insurance, history of anxiety, gestational age, gravida, number of scores, and total fentanyl administered.

Model	Initiative	N	Risk Ratio	95% Confidence Interval
Model 1: Unadjusted	Pre-initiative	146	Ref	-
	Post-initiative	216	0.95	0.85-1.07
Model 2: Adjusted	Pre-initiative	146	Ref	-
	Post-initiative	216	0.96	0.85-1.08

## Discussion

Summary

Our quality improvement initiative using a standardized sedation options counseling guide to provide patient-focused counseling decreased pain, but not anxiety, during abortion procedures. As a post hoc analysis, the results of this study provide insight into pain management in the context of clinical care in busy health centers.

Interpretation

Because all patients included in the analysis received moderate sedation, and there was no change in the sedation options available and no difference in total fentanyl doses between the two groups, the statistically significant change in procedural pain scores was likely associated with the patient-focused counseling initiative. This study adds a standardized sedation options counseling guide to the list of options shown in other studies to reduce pain management during procedural abortions [1-3]. While no studies have previously considered counseling as an opportunity to advance abortion pain management, we can now consider it as a method to improve patient care for the future.

Limitations

Because we were assessing a quality improvement initiative in its real-world clinical practice setting, missing data was a limitation. However, the amount of missing data was equivalent between the pre-initiative and post-initiative groups and unlikely to have biased our results.

The quality improvement initiative took place in three outpatient health centers in Northern New England and does not necessarily reflect how the initiative might be used or received in health centers in other parts of the country. Due to its geographic limitations, racial diversity was limited. However, the study included all procedures with moderate sedation and complete data performed at the health centers to eliminate sampling bias. Assessing the use of patient-focused counseling in other settings is warranted.

The lack of patient feedback on the counseling limits the assessment of this quality improvement initiative. Staff feedback was limited to anecdotal feedback and those who spoke up during the open forum follow-up, so a more structured process to collect feedback may have been helpful. The counseling guide developed and implemented was a first draft, and as with all quality improvement initiatives, we plan to adjust the process as needed in the future.

Standardizing sedation options counseling using the patient-focused guide should reduce variability in counseling delivery, but variability among counseling delivery may persist as a limitation.

While the study faces limitations such as missing data, a lack of geographical and racial diversity, a lack of formalized patient and staff feedback, and potential counseling variability, these types of limitations are to be expected in an intervention designed as a local quality improvement initiative rather than a research study [15]. Our intention was to improve counseling at our health centers, not necessarily to create generalizable knowledge. However, other health centers may benefit from our counseling guide because of the evaluation of its use in real-world clinical practice.

## Conclusions

Health center staff frequently contribute to continuous quality improvement by identifying needs and finding ways to address them, often without the capacity or intention to evaluate the interventions they implement. While they may recognize that the guide meets their immediate intended need, any additional unintended benefits on a larger scale cannot be determined without further analysis. Studying quality improvement interventions is essential to discover the intentional and unintentional impacts that interventions may have.

The clinical implications of this study are considerable, as health centers can adjust counseling practices to decrease the odds of procedural pain without any likely side effects for patients. This adjunct to established pain management options is a simple improvement on current practice. We replaced our previous practice of counseling rather than adding additional interventions, making the use of the guide practical and sustainable in the long term. The findings and conclusions in this article are those of the authors and do not necessarily represent the views of Planned Parenthood Federation of America, Inc.

## Figures and Tables

**Figure 4:**
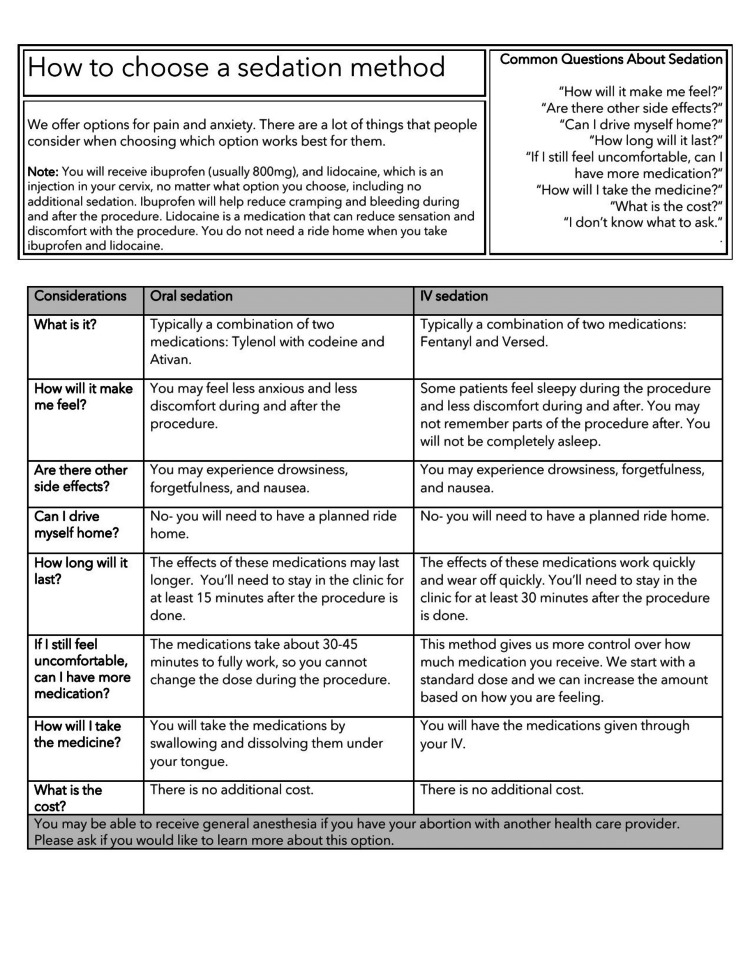
Standardized Patient-Focused Sedation Options Counseling Guide
